# An Innovative Non-Invasive Method for Early Detection and Monitoring of Acute Compartment Syndrome

**DOI:** 10.3390/jpm14050477

**Published:** 2024-04-29

**Authors:** Razvan Tudor Tepordei, Carmen Lacramioara Zamfir, Alin Horatiu Nedelcu, Ovidiu Gabriel Avadanei, Tudor Cozma, Ovidiu Alexa, Manuela Ursaru, Lacramioara Perianu, Iuliana Magdalena Starcea, Ileana Ioniuc, Vasile Valeriu Lupu, Ancuta Lupu

**Affiliations:** 1 Department of Morpho-Functional Science I, Discipline of Anatomy, “Grigore T. Popa” University of Medicine and Pharmacy, 16 Universitatii Street, 700115 Iasi, Romania; razvan.tepordei@umfiasi.ro (R.T.T.); carmen.zamfir@umfiasi.ro (C.L.Z.); lacramioara.perianu@umfiasi.ro (L.P.); 2 Department of Orthopaedics and Traumatology, “Sf. Spiridon” Emergency Universitary Hospital, 700115 Iasi, Romania; tudor.cozma@umfiasi.ro (T.C.); ovidiu.alexa@umfiasi.ro (O.A.); 3 Department of Physics, Al. I. Cuza University, 11 Carol I Bv, 700506 Iasi, Romania; minu@uaic.ro; 4 Department of Surgical Sciences II, “Grigore T. Popa” University of Medicine and Pharmacy, 16 Universitatii Street, 700115 Iasi, Romania; 5 Department of Surgical Sciences I, “Grigore T. Popa” University of Medicine and Pharmacy, 16 Universitatii Street, 700115 Iasi, Romania; manuela.ursaru@umfiasi.ro; 6 Department of Mother and Child, “Grigore T. Popa” University of Medicine and Pharmacy, 16 Universitatii Street, 700115 Iasi, Romania; iuliana.starcea@umfiasi.ro (I.M.S.); ileana.ioniuc@umfiasi.ro (I.I.); vasile.lupu@umfiasi.ro (V.V.L.); ancuta.ignat1@umfiasi.ro (A.L.)

**Keywords:** trauma, acute compartment syndrome, intracompartmental pressure, thermic gradient, temperature sensor

## Abstract

**Background:** Acute compartment syndrome is a major surgical emergency with complex pathophysiology and a highly unpredictable pattern of evolution. We hypothesized that the onset of acute compartment syndrome of the leg or forearm is associated with variations in the surface temperature of the distal segment (foot or hand) with a distinct pattern, which acts as an early warning sign. **Materials and Methods:** We developed a monitoring device that consists of two thermic sensors attached to a modular limb splint, which continuously measure the temperature difference between the proximal and distal regions of the limb (i.e., arm–hand, thigh–foot). Firstly, we investigated both the arm–hand and thigh–foot temperature gradients of hospitalized patients’ healthy limbs (43 patients, 56 upper limbs, 64 lower limbs) in order to establish a baseline. Secondly, we examined the correlation between the thermic gradients and intracompartmental pressure values in compartment syndrome limbs (20 patients, 6 upper limbs, 14 lower limbs). **Results:** For the control group, the mean values for the normal limb thermic gradients were −0.17 °C for the upper limbs. and 0.03 °C for the lower limbs. In the impending compartment syndrome group (defined by intracompartmental pressure values), the mean index was −0.38 °C. In the fully developed compartment syndrome group, the mean value was 4.11 °C. **Discussions:** Analysis was performed using the ANOVA one-way statistical method. This showed significant differences between the compartment syndrome group and the impending and control groups. A decreasing trend in the thermic gradient in patients with impending compartment syndrome compared with the control group was noted. **Conclusions:** The thermic gradient of limbs presenting signs of impending compartment syndrome decreases as a result of the increased temperature of the distal segment. This pattern can be used as an early diagnostic method for acute compartment syndrome. This technique is non-invasive and bears no risk to the patient, allowing facile continuous monitoring during immobilization.

## 1. Introduction

Acute compartment syndrome (ACS), regardless of its localization, is a severe complication, with significantly debilitating potential. Muscle and nerve ischemia results from an imbalance between the perfusion pressure and the metabolic demands of a tissue. Its early diagnosis is difficult, especially in children or unresponsive patients, and its evolution is highly unpredictable. In this context, the methods for early recognition of ACS are still a source of debate, leading to treatment delays and both local and systemic complications. The prognosis can be only belatedly expressed. Because of its heterogeneous etiology and its complex pathology, which is still incompletely understood, this syndrome represents a redoubtable condition that even with adequate treatment may have a fatal outcome [1]. In stark contrast with these multiple considerations, the sole recommended treatment is decompressive fasciotomy [2,3]. 

Therefore, the problem is not in choosing an appropriate method of treatment, but in the recognition of acute compartment syndrome at its onset. Outcomes are closely related both to the promptness of the diagnosis and to the period between injury and treatment. The orthopedic clinician must have a high index of suspicion in all trauma cases, especially in comatose or pediatric patients. The gold standard for diagnosis is the direct measurement of intracompartmental pressure. This is achieved through invasive techniques that offer only a brief look into the evolution of the disease. The risk of permanent muscular and nervous damage versus an almost complete recovery is firmly correlated with the promptness of therapeutic decisions.

Our diagnosis method provides a solution to the problem of identifying the onset of local microcirculation alteration characteristics associated with ACS. The purpose of our research was to investigate the hypothesis that an increased temperature difference between the proximal and the distal regions of a limb has real diagnostic significance. To measure this temperature difference, we used a modular splint with thermic sensors attached to the proximal and distal segments. The device we propose here has a double function: to immobilize a fractured limb, and to realize continuous, non-invasive monitoring of the thermic gradient of the limb. The results produced can be useful for clinicians, offering an alternative to invasive pressure determination.

## 2. Materials and Methods

To investigate our hypothesis, we first established a baseline by determining the normal values of the thermic gradient of both the upper limb (temperature difference between the arm and hand, upper limb index) and lower limb (temperature difference between the thigh and the foot, lower limb index) in the healthy, uninjured limbs of hospitalized patients who had been admitted for surgical treatment of various musculoskeletal lesions. Consequently, we examined the correlations between intracompartmental pressure values and the value of the thermic index reported in the limbs affected by acute compartment syndrome. 

In order to identify the normal values of the thermic gradients in hospitalized patients, we examined 43 patients from the Orthopedics and Traumatology Clinic at “Sf. Spiridon” Emergency University Hospital, Iasi. The demographic details of these patients were as follows: 26 men and 17 women; 20 pre-operative and 23 post-operative; aged between 19 and 57 years; 56 healthy upper limbs, and 64 healthy lower limbs. These patients formed the control group. They were hospitalized for acute injuries of the locomotor system, excluding polytrauma and coma. Patients with associated pathologies that may have caused variations in skin temperature in the extremities were excluded. Another reason for excluding patients with other pathologies was their use of medication that may have modified the recorded surface thermal values of the extremities.

The patients in the post-operative period did not receive drugs likely to impair blood circulation in the limbs. All of the patients had temperatures measured at an interval of at least 72 h post-operatively in order to minimize the effects of anesthetic drugs administered during surgical intervention.

For each patient, the local cutaneous temperature was measured from a contralateral healthy and uninjured limb with a Thermoval Clinical Thermometer (Paul Hartmann AG, Heidenheim, Germany). In the upper limbs, we measured the temperatures between two points located as follows: on the anterior face of the arm midpoint between the acromion and the cubital fossa crease, and on the dorsum of the hand at the second interosseous space. The two measurement points corresponding to the lower limbs were: on the anterior face of the thigh midpoint between the anterior superior iliac spine and the base of the patella, and on the dorsum of the foot at the second interosseous space. Using these benchmarks, we avoided the regions superposed to important blood vessels, and those isolated from the environment by the dorsal decubitus of the patients. The limbs were exposed to the standard environmental conditions at least 10 min before the measurements were taken.

**Figure 1:**
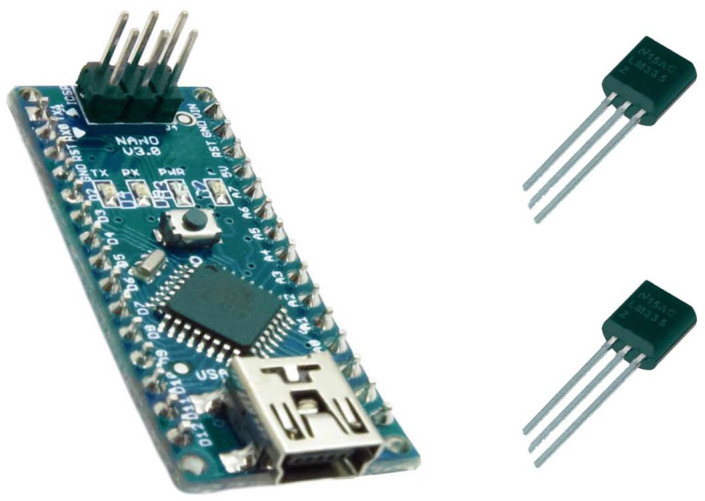
The Arduino microcontroller and the sensors.

To continuously monitor the temperature and to easily calculate the proximal–distal thermal gradient, we used an Arduino Kit µC ATMEGA328 (BCMI Labs, Monza, Italy), which is an open-source programmable electronic platform, and two low-cost and low-consumption thermal sensors, LM335 (STMicroelectronics, Plan-les-Ouates, Switzerland) (Figure 1). The Arduino microcontroller presented the registered thermal values and the difference between them on an RT162 LCD (Nortech Engineering Inc., Port Charlotte, FL, USA) display in real time, using the output voltages of the two sensors.

If the thermal gradient was more than 2 °C (a value that can be easily adjusted), the microcontroller sent a 1 KHz signal to a 5V Buzzer via a 2N7000 transistor (STMicroelectronics, Plan-les-Ouates, Switzerland), which provided the necessary current required by the Buzzer. We also used a 5V 1A LM7805 stabilizer (STMicroelectronics, Plan-les-Ouates, Switzerland) in order to ensure that the entire device would work properly, even if the voltage supply was not constant.

**Figure 2:**
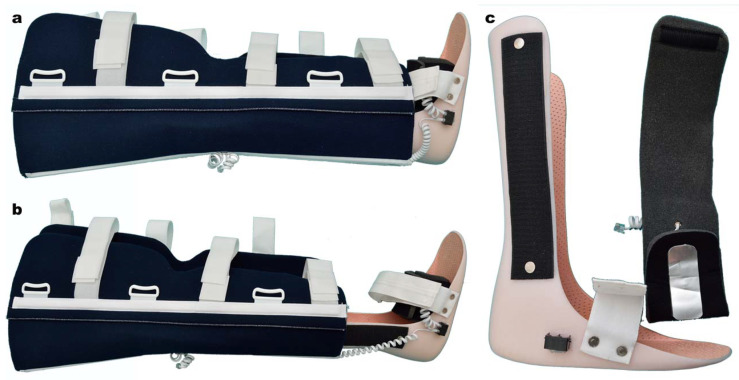
Lower limb modular splint. (**a**,**b**) length control; (**c**) distal thermic sensors.

**Figure 3:**
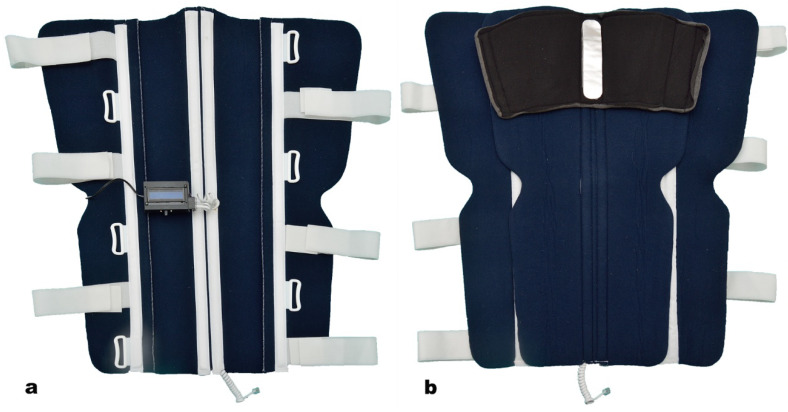
Lower limb modular splint. Proximal component. (**a**) Outer aspect and the electronic device. (**b**) Internal surface with thermic sensor.

The device was attached to a modular splint for limb immobilization (Figure 2). The sensors were placed in the following manner: one on the proximal component of the splint, and the other on the distal component (Figure 3). By applying the splint on the affected limb, the thermic sensors contacted the tegument of the proximal and distal regions, thus detecting the temperature difference between the two segments.

As an alternative, the sensor pair was able to be utilized independently, separate from the modular splint, incorporated in a plaster cast. In this simplified version, the device lacked the digital display, but gained a high degree of autonomy, operating in a continuous manner with a single 9V battery for almost 14 days. The complete version, because of its higher power demands, used a 12 V wall adapter.

The splint contained two components. The proximal component was made of reinforced neoprene with aluminum bars (Figure 3). The distal component was made of a thermoform material, using a universal left-right mold, with fixed external Velcro bands, which allowed connection with the proximal component, and length control (Figure 2). RJ11 connectors were attached to both components. 

**Figure 4:**
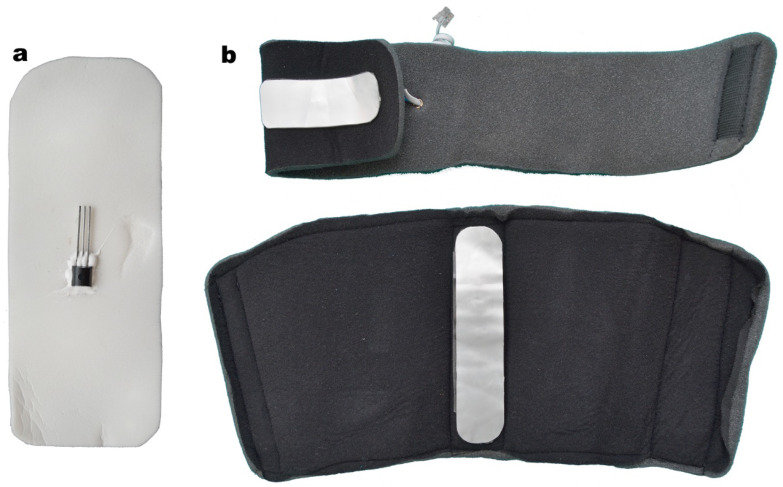
(**a**) Thermic sensor attached to the metallic foil. (**b**) Thermic sensors for the distal and proximal component.

We fixed the thermic sensors to a flexible metal plate using a thermoconductive paste, similar to the type used in electrosurgery, which is well tolerated on skin contact (Figure 4).

To correlate the intracompartmental pressure values with the thermic gradient values, we included 20 patients, 15 males and 5 females, aged between 19 and 62, with either impending or fully developed ACS. Of this group, 11 patients had leg fractures (6 ACS and 5 impending ACS), 3 had tibia plateau fractures (1 ACS and 2 impending ACS), 3 had distal radius fractures (2 ACS and 1 impending ACS), 1 had an olecranon fracture and ACS, and 2 had grade III elbow sprains (1 ACS and 1 impending ACS). We excluded patients with skin lesions on the proximal and distal segments. The inclusion and exclusion criteria are presented in Table 1.

We used a compartment pressure measuring system (CPMS—Mammendorf Institute for Physics and Medicine, Mammendorf, Germany) in continuous mode to assess intracompartmental pressure and to identify ACS cases, following the principle of the pressure gradient ∆P (the difference between diastolic systemic pressure and the intracompartmental pressure). 

**Figure 5:**
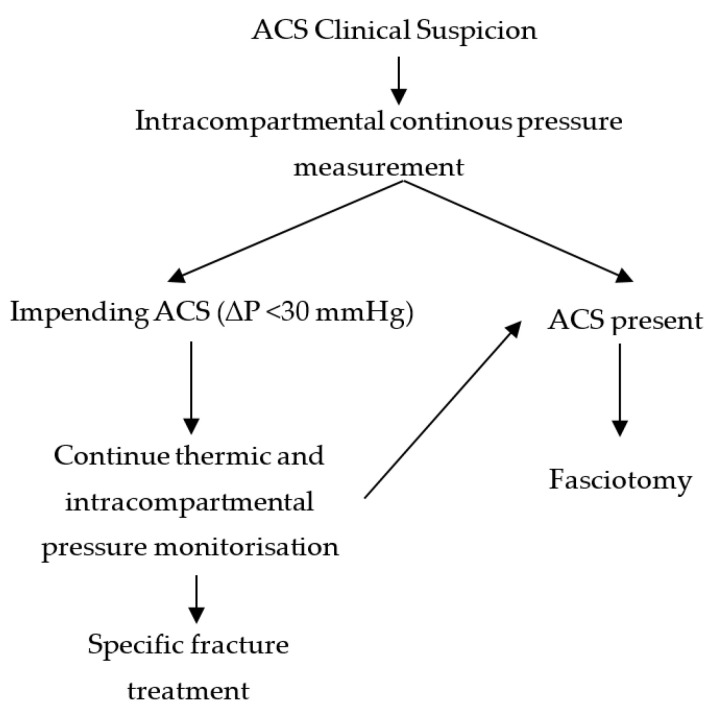
Therapeutic protocol diagram.

Each patient with leg/forearm musculoskeletal lesions was assessed clinically for signs of ACS (pain disproportionate to the lesion, pain with passive mobilization, and tension oedema of the affected segment). For those cases which raised reasonable suspicion, we continuously (24/24) measured the intracompartmental pressure of the affected limb, and applied the thermic monitoring splint (Figure 5). 

**Figure 6:**
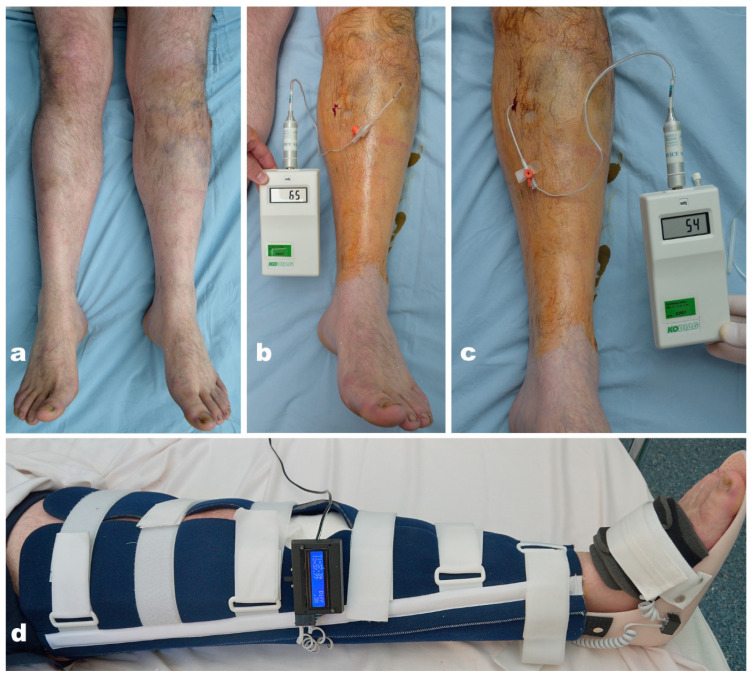
(**a**) Fracture of the left tibia plateau; ACS suspected. (**b**,**c**) Increased values of intracompartmental pressure in the anterior and posterior compartments (BP = 151/101 mmHg, ∆P > 30); impending ACS. (**d**) Immobilization with the thermic monitoring splint (∆T = 0.38 °C).

All patients with values of ∆P < 30 mmHg were considered to have ACS, and those with ∆P between 31 and 40 mmHg were considered to have impending ACS. All lower limb fractures with increased intracompartmental pressures were immobilized with the continuous monitoring splint, both in the pre- and post-operative periods (Figure 6). All ACS cases were surgically confirmed.

Statistical analysis was performed with SPSS 17 software, using an independent samples T-test, paired samples T-test, Pearson bivariate correlations, Spearman correlations, and ANOVA one-way.

## 3. Results

The main characteristics of the control and ACS groups were studied (Table 2). Results showed that the control group had an average hand temperature (M = 36.65) significantly higher than the average arm temperature (M = 36.50; t(63) = −3.53, *p* < 0.01), and that this difference was present both in pre-operative (M_arm_ = 36.50, M_hand_ = 36.65, t(17) = −2.38, *p* < 0.05) and post-operative patients (M_arm_ = 36.44, M_hand_ = 36.64, t(38) = −3.71, *p* < 0.01). There were no significant differences between the temperatures measured in the lower limb. In the general sample, the average upper and lower limb temperature gradients were not significantly different, but in the post-operative condition, the results showed a lower limb average gradient (M = 0.03) significantly higher than the upper limb gradient (M = −0.17, t(21) = 2.43, *p* < 0.05).

**Figure 7:**
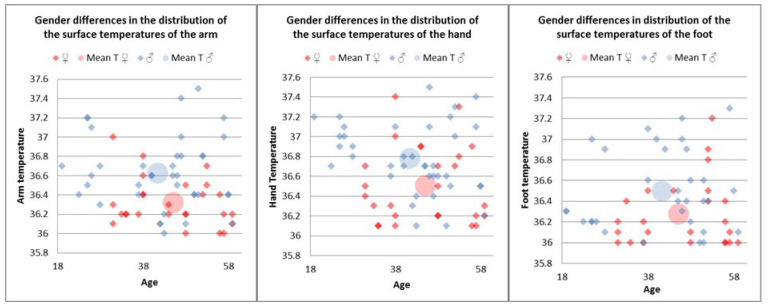
Gender differences in distribution of the surface temperatures of the arm, hand, and foot in the control group.

Gender significantly influenced the temperatures of the upper limb, with male patients having higher arm (M = 36.63) and hand (M = 36.76) temperatures than female patients (M_arm_ = 36.31, t(62) = 4.06, *p* < 0.01 and M_hand_ = 36.50, t(62) = 2.88, *p* < 0.01). Similar gender differences were found in average foot temperatures, with significantly higher values in men (M = 36.47) than women (M = 36.27, t(53) = 2.08, *p* < 0.05) (Figure 7).

The results also indicated within-gender differences between temperatures in the upper limb, with the arm temperature being significantly lower than the hand temperature in both male (M_arm_ = 36.63, M_hand_ = 36.76, t(27) = −2.44, *p* < 0.05) and female patients (M_arm_ = 36.31, M_hand_ = 36.50, t(25) = −2.52, *p* < 0.05). Moreover, in female patients, the lower limb average temperature index (M = 0.05) was significantly higher than the upper limb index (M = −0.15, t(16) = 2.24, *p* < 0.05).

We also found a marginal positive correlation (r = 0.29, *p* = 0.06) between the temperature indexes of the upper and the lower limb, meaning that an increase in one index tends to be associated with an increase in the other index. A closer examination of the results shows that this correlation was statistically significant and stronger in the post-operative patients (r = 0.47, *p* < 0.05), while in the pre-operative condition, it remained weak and nonsignificant (r = 0.21, *p* > 0.05). For the pre-operative condition only, age was significantly negatively correlated with both arm (r = −0.75, *p* < 0.01) and hand (r = −0.81, *p* < 0.01) temperatures, meaning that with increasing age, their values tended to decrease.

**Figure 8:**
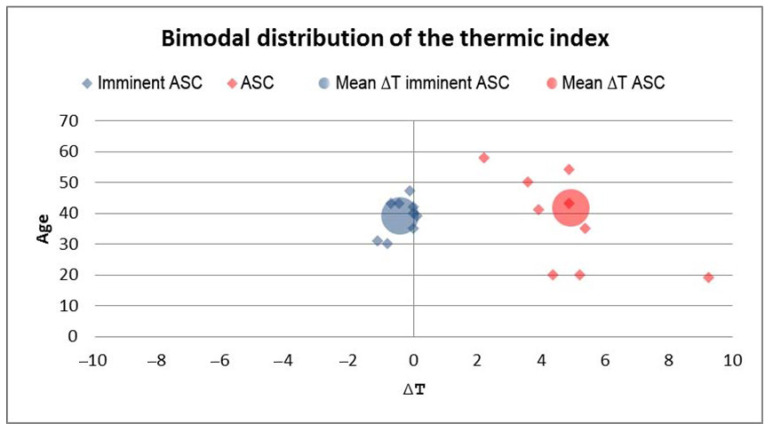
Bimodal distribution of the thermic index in patients with impending ACS and present ACS. The impending ACS group had a negative thermic index mean compared to the ACS group.

Regarding the ACS group, we observed considerably increased thermic index values in patients with manifested ACS. In the impending ACS group, we detected thermic indexes close to zero and negative, with the distal segment temperature being higher than the proximal (Figure 8).

Using the ANOVA one-way statistical method, we observed that there were significant differences regarding the thermic indexes of the patients, depending on the absence, imminence, or presence of ACS (F(2, 137) = 114.32, *p* < 0.01). Means, standard deviations, and the number of patients for each group are presented in Table 3.

**Figure 9:**
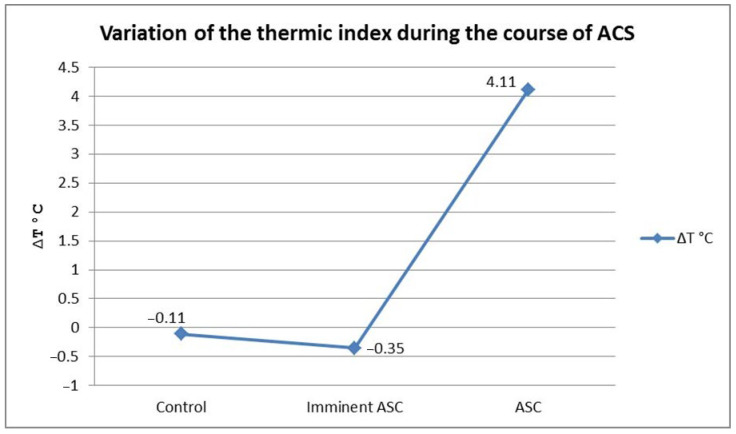
The dynamic of ACS evolution. Slight drop of thermic index in the impending ACS group as a result of distal segment surface temperature increase, followed by a marked increase in thermic index in the ACS group.

To verify the presence of significant differences between groups of patients, we applied the post-hoc Bonferroni test. Results showed that the thermic index values in patients with ACS were significantly increased compared with those of the patients with impending ACS (Bonferroni = 11.15, *p* < 0.01), and with those of the control group, respectively (Bonferroni = 15.07, *p* < 0.01). Although not statistically significant, we found a tendency towards a decreased thermic index in patients with impending ACS compared with the control group (Figure 9).

Another element of our study was to assess any statistically significant differences between the thermic indexes of the patients according to the number of affected compartments. The results indicated an important effect of this variable on thermic index variability (F(4, 127) = 228.34, *p* < 0.01). Means, standard deviations, and the number of patients for each group are presented in Table 4. The Bonferroni post-hoc test revealed the following differences between groups: the thermic index of the control group was significantly smaller compared with the patients with two (Bonferroni = 9.60, *p* < 0.01), three (Bonferroni = 18.83, *p* < 0.01), and four (Bonferroni = 22.59, *p* < 0.01) affected compartments, respectively. Those with one affected compartment had a significantly lower thermic index than those with two (Bonferroni = 7.85, *p* < 0.01), three (Bonferroni = 12.34, *p* < 0.01), and four (Bonferroni = 17.84, *p* < 0.01), respectively.

**Figure 10:**
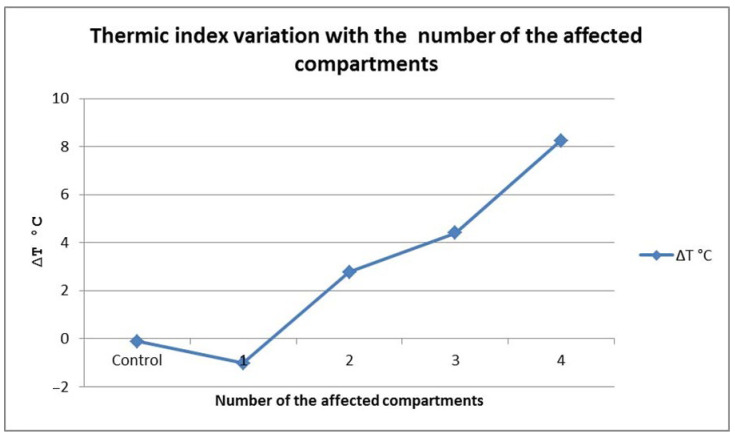
∆T variation with the number of affected compartments.

The values of the thermic index were also lower in patients with two affected compartments compared with the patients with three (Bonferroni = 4.31, *p* < 0.01) and four (Bonferroni = 11.41, *p* < 0.01) affected compartments, with similar differences being present between the last two groups (Bonferroni = 8.72). As Figure 10 shows, when compared to the control group, the thermic index tended to decrease in patients with only one affected compartment (though this was not statistically significant). After that, the thermic index values followed an ascendant trend in direct relation with an increasing number of affected compartments.

In order to observe the relationship between the thermic index and the pressure gradient in all four compartments of the upper limb, we calculated the ρ Spearman correlation coefficient. The results showed that in the anterior (ρ = −0.77, *p* > 0.05), lateral (ρ = −0.71, *p* > 0.05), and deep (ρ = −0.77, *p* > 0.05) compartments, respectively, there were strong negative correlations between the two factors, meaning that as the thermic index became higher, the pressure gradient tended to decrease. However, there was an exception; in the pronator quadratus compartment, the correlation was positive (ρ = 0.48, *p* > 0.05), with high thermic gradient values being associated with high values of the pressure gradient. Although these correlations did not meet the statistical significance criteria, most probably because of our reduced sample, they were high enough to have practical significance in diagnosis of the syndrome, with the value of the R2 determining coefficient being around 0.45 (indicating that almost 45% of the pressure gradient variance can be explained by the variability of the thermic index). 

Similar analysis conducted for the four compartments of the lower limb revealed the same negative, strong, and this time statistically significant, correlational pattern between the values of the thermic index and those of the pressure gradient in all of the compartments, meaning that as the thermic index became higher, the pressure gradient tended to decrease. The strongest correlation appeared in the lateral compartment (ρ = −0.82, *p* < 0.01), followed the anterior (ρ = −0.77, *p* < 0.01), deep posterior (ρ = −0.73, *p* < 0.01) and superficial posterior (ρ = −0.67, *p* < 0.01) compartments, respectively.

## 4. Discussion

In healthy, non-hospitalized subjects, normal temperatures of the foot and hand vary widely with the environmental temperature (from 15.9 °C during winter to 37.5 °C in summer) [4] and subject activity level [5,6,7]. Nardin et al. [4] examined variations in the surface temperature of the foot in 39 healthy subjects, aged between 18 and 65, during daytime and nighttime. They reported a mean temperature of the foot of 30.6 °C during the daytime and 34 °C at night, but this varied widely in relation to the ambient temperature, from 15.9 °C in winter to 37.5 °C during summer. Ceron et al. [5] investigated variations in hand temperature in workers in cold (13 °C) and warm (23.9 °C) environments and obtained a wide distribution of surface temperatures which were directly correlated to environmental conditions. Our data indicated a quasi-constant temperature of around 36.5 °C. This discrepancy can be explained by the fact that our subjects were in-patients with reduced activity levels; the majority of them were confined to bed, thus eliminating activity level from equation. In addition, the hospital ambient temperature is constant (24 ± 0.5 °C); hence, variations in body temperature cannot be linked to variations in the ambient temperature. 

To our knowledge, there are no descriptions in the literature of the investigated thermic gradients (thigh–foot and arm–hand) in healthy limbs, which prompted us to investigate further to obtain a baseline. During our study, we obtained gradient values close to zero (−0.17 °C arm–hand thermic gradient and −0.06 °C thigh–foot thermic gradient). Hence, we considered these values as baseline.

The value of 30 mmHg for the intracompartmental pressure was considered an absolute indication for decompressive fasciotomy in order to prevent the evolution of compartment syndrome [8], although many authors admit that this value is heavily influenced by the hemodynamic status of the patient [9,10,11]. Tissue tolerance to increased pressure values, such as those present in compartment syndrome, varies widely depending on three factors: the specific effect on the local blood flow, the metabolic needs of the tissues, and the duration of the pressure. The effects of these factors are nuanced by the clinical characteristics of the patient, including the presence of hypotension, shock, arterial spasm, and prone position of the limb. Thus, a single absolute intracompartmental pressure value cannot be applied indiscriminately as a clear indication for fasciotomy. For this reason, Whiteside proposed the use of the difference between the blood diastolic pressure and the intracompartmental pressure as a reference [12,13]. The value of this pressure gradient for the indication of decompressive fasciotomy is 30 mmHg, and this correlates much better with clinical practice. 

In simple trauma fractures (without ACS), there is a local hyperemia explained by the nonspecific inflammatory reaction, providing local defense. This hyperemia caused by local vasodilation is clinically expressed by an increase in the local temperature, without alterations of the distal segment [14,15,16]. In ACS, regardless of the involved mechanism, the common factor is a decrease in tissue perfusion through a decrease in the local blood flow, as a consequence of increased intracompartmental pressure [17,18]. Arterial pressure, venous pressure, vascular resistance, and interstitial pressure represent essential factors in blood pressure [19,20]. The arterio-venous gradient is directly involved in the maintenance of normal blood pressure values. This gradient is defined as the difference between arterial and venous pressure. Conditions that decrease arterial blood pressure (hypovolemia, shock, and elevated position of the anatomic segment) or raise the venous pressure will reduce the arterio-venous gradient and the perfusion rate [21]. This theory of the arterio-venous gradient explains, but in an incomplete manner, the appearance and evolution of ACS [22]. At the same time, it can explain the increase in the distal segment temperature detected by us in the early stages of ACS. 

Katz et al. [23] hypothesized that ACS is associated with a decrease in the cutaneous temperature of the distal segment from the injury. They investigated this hypothesis in the lower limb, using an infrared video camera. The results showed statistically significant differences between the temperatures of the thigh and the foot (thigh–foot index) in ACS legs (8 °C), demonstrating the usefulness of this method in the detection of this syndrome.

Distal thermic cutaneous alterations and the evolution of ACS have an interdependent dynamic, which allowed us to identify two distinct groups, one with impending ACS, and one with present ACS.

We have identified a pattern in the dynamics of ACS onset: the average of the thermic gradient in patients with impending ACS decreases and becomes negative (mean ∆t = −0.38 °C) because of the increasing temperature of the distal segment. After that, along with the evolution of ACS, this gradient increases and becomes positive (mean ∆t = 4.11 °C) in accordance with a drop in the cutaneous temperature of the foot or hand. This initial increase in the distal segment temperature acts as an early warning signal and can be used to identify cases of impending ACS. 

The number of affected compartments also controls the distal segment temperature. When only one compartment was affected, the distal segment temperature was increased, but we were not able to find a statistic significant difference between this group and the control group. During the successive recruitment of all of the neighboring compartments, the distal segment temperature decreased; in those cases, the measurement of the thermic gradient became useful to define the evolutive stages of ACS.

The triggering factor is represented by tissue edema, in the context of two main physiopathological mechanisms that reduce the transmural pressure gradient: the decrease in arterial-capillary flow and the presence of a non-specific local inflammatory reaction, secondary to the trauma. A vicious cycle of edema-ischemia-pressure is created, with progressive evolution, which can successively affect all of the osteofascial compartments of an anatomical segment. The different weight of these two mechanisms in a given case will give the clinical picture distinct characters of different intensities and durations, making it difficult to identify risk factors and stage the disease. None of the theories that have tried to explain the pathophysiological mechanisms of compartment syndrome have provided a definitive verdict. Rather, the mechanisms are intricate and complement each other, making a characterization with predictive value on the evolution and prognosis of this disease impossible.

There has been growing interest in the use of NIR spectroscopy (near-infrared spectroscopy), which is based on specific changes in the transmission and absorption of the near-infrared electromagnetic spectrum by the oxygen saturation level of hemoglobin. Variations in tissue hemoglobin saturation percentage lead to variations in the absorption of infrared radiation. One of the limitations of this method is that its tissue penetration does not exceed 3 cm [24,25], making it useful only for measuring superficial compartments. Rostosky et al. [26] measured subcutaneous adipose tissue thickness at the ankle in 50 patients with severe calf injuries by ultrasonography. They did not notice differences between injured and healthy limbs (6.98 mm vs. 7.06 mm) and did not obtain thicknesses of subcutaneous adipose tissue greater than 2 cm in any patient, validating the usefulness of this method.

It should be noted that in uncomplicated leg trauma with acute compartment syndrome, local hyperemia due to the non-specific inflammatory process will increase the percentage of oxygenated hemoglobin, a phenomenon objectified by Shuler and colleagues [24], who compared 26 patients with unilateral tibial fractures with 25 healthy subjects, spectroscopically measuring the percentage of oxygenated hemoglobin in all four compartments of the calf. They obtained values that showed that the average hemoglobin oxygen saturation increased by 15.4% in the compartments of the anatomical segment with fracture injuries as compared to the contralateral limb and healthy subjects. More recent studies [14,27,28] have aimed to objectify the perfusion deficit by using NIR spectroscopy in acute compartment syndrome located at the level of the calf. In all of these patients, invasive determination of the intracompartmental pressure as well as spectroscopic measurement of the contralateral intact leg were performed. At the level of the affected compartments (with high pressure values and differences between diastolic and intracompartmental pressure < 10 mmHg), the percentage of oxygenated hemoglobin was shown to have decreased by approximately 10% compared to the contralateral limb, thus demonstrating a positive correlation between the differences in the spectroscopic values of the unaffected and affected compartments and the pressure gradient ∆P.

Thus, a phenomenon similar to the one we identified in our thermal and pressure correlation study appears: initially, local hyperemia increases the temperature of the affected segment, and then the perfusion deficit leads to a decrease in temperature. In this light, continuous temperature monitoring of the distal segment is of real use for the detection of impending acute compartment syndrome, and represents a much cheaper and more accessible technique than NIR spectroscopy.

The design and execution of a universal splint that correctly immobilizes a fracture is difficult, having to take into account the differences in the dimensions of the limbs, both in length and in diameter. We opted for the principle of modularity. because even if this increases the complexity, it provides a better adaptation to size variations in the anatomical segments involved. The contact of the splint with the integument must be as intimate as possible, especially at the level of the components containing the temperature sensors, in order not to introduce errors in the measurement of thermal values. This was achieved through a compromise between universality and stability. Any form of immobilization, to be perfectly stable, must be personalized to the patient, and any method of universal immobilization will have to give up customization in favor of adaptability. Thus, the principle of modularity was applied, combining the two points of view located at opposite ends.

The presence of acute or chronic skin conditions can prevent the device from working optimally, given that the contact point of the sensors with the skin cannot be changed. In the case of open fractures, this impediment does not exist, because the injuries are at the level of the intervening segment (leg or forearm), and not at the level of the proximal (thigh or arm) or distal (leg or hand) segments where the temperature sensors are placed. Chronic vascular trophic skin conditions can lead to erroneous temperature values by changing the values of the distal segment. Usually, these conditions are found in the elderly (over 60 years old), in whom compartment syndrome has a very low incidence rate. In young people, the presence of these chronic conditions is exceptional, so this associated pathology is not an impediment. Skin lesions of any nature, acute or chronic, located at the sensor contact areas (second interosseous space on the dorsal side of the foot, second interosseous space on the dorsal side of the hand, anterior face of the arm, and anterior face of the thigh) is a contraindication for temperature measurement at these levels. In this case, the sensors of the splint can be disassembled, and applied separately to regions with intact skin.

The limitations of our study come from the fact that acute compartment syndrome is a rare complication, and consequently, the number of selected cases was relatively small. Another limitation is the fact that we used a control group consisting of healthy, uninjured limbs (the contralateral uninjured limb of a patient with an uncomplicated fracture). The literature agrees that in response to injury, the temperature of the involved segment rises. To our knowledge, in a trauma uncomplicated by ACS, local temperature increase (ROI) does not involve the temperature of the distal or proximal limb segment. Moreover, our study did not determine the absolute thermal values, but the variation in the thermal gradient during continuous monitoring between the extremities of the affected limb (thigh–foot and arm–hand). A higher number of cases, perhaps using a multicentric study, should be employed to achieve better statistical confidence.

## 5. Conclusions

The initial stages of ACS can be detected using a dynamic measurement of thermic gradient, which signals the onset of ACS by the recruitment of the first compartment. Our device acts as an early warning alarm, identifying the beginning of ACS.

Despite the abovementioned limitations, we believe our method to be valid and useful in clinical practice. Moreover, with the development of artificial intelligence, we will be able to create a connection between AI and our medical device made. Future research should extend the investigation, testing the scientific reliability of both our proposed alternative diagnostic method and our monitoring device.

## Figures and Tables

**Table 1:** Inclusion and exclusion criteria.

Inclusion Criteria	Exclusion Criteria
1. Age over 18 years	1. Age under 18 years
2. Acute injuries of the locomotor system	2. Medication that can modify the thermal values
3. Only one limb affected	3. Skin lesions on proximal and distal segments.

**Table 2:** Main characteristics of the explored groups.

Characteristics	Control Group (n = 43)	Explored Group (n = 20)
Mean age	41.84 years	40.45 years
Male/Female	1.5/1	3/1
Upper limb	64	6
Lower limb	56	14
Diastolic blood pressure	86.4 mmHg	81.1 mmHg
Environmental temperature	24 ± 0.5 °C	
Patients’ mean temperature	36.4 (36–37.5)	36.9 (36.6–37.6)
Time from fracture	48–96 h	6–72 h
Associated pathology	6	2
Associated fractures	44	4
Open fractures	0	1
Craniocerebral trauma	0	1
Coma	0	1
Pre-surgery	20	20
Post-surgery	23	0

**Table 3:** Mean thermic index distribution in the control group, impending, and fully developed ASC groups.

ASC/Impending/Control	n	Thermic Index	
		M	SD
ASC	11	4.11	3.02
Impending	9	−0.35	0.51
Control	120	−0.11	0.37

**Table 4:** Subject distribution by the number of the affected compartments.

Number of Affected Compartments	n	Thermic Index	
		Mean	SD
Control	120	−0.11	0.37
1 compartment	2	−1.02	0.85
2 compartments	3	2.77	1.01
3 compartments	5	4.41	1.86
4 compartments	2	8.25	1.44

## Data Availability

The data presented in this article are available on request from the corresponding author.
